# Gliadin Intake Causes Disruption of the Intestinal Barrier and an Increase in Germ Cell Apoptosis in A *Caenorhabditis Elegans* Model

**DOI:** 10.3390/nu11112587

**Published:** 2019-10-27

**Authors:** Hyemin Min, Ji-Sun Kim, Jiyun Ahn, Yhong-Hee Shim

**Affiliations:** 1 Department of Bioscience and Biotechnology, Konkuk University, Seoul 05029, Korea; 2 Division of Nutrition and Metabolism Research, Korea Food Research Institute, Jeollabuk-do 55365, Korea; sunflower418@hanmail.net (J.-S.K.); jyan@kfri.re.kr (J.A.)

**Keywords:** gluten toxicity, gliadin intake, reactive oxygen species, intestinal barrier, germ cell apoptosis, *Caenorhabditis elegans*

## Abstract

Gliadin is a major protein component of gluten and causes gluten toxicity through intestinal stress. We previously showed that gliadin intake induces oxidative stress in the intestine and reduces fertility in a *Caenorhabditis elegans* model. To elucidate the possible link between intestinal stress and reproduction, changes in the intestine and germ cells of *C. elegans* after gliadin intake were examined at the molecular level. Gliadin intake increased reactive oxygen species (ROS) production in the intestine, decreased intestinal F-actin levels, and increased germ cell apoptosis. These gliadin-triggered effects were suppressed by antioxidant treatment. These results suggest that ROS production in the intestine induced by gliadin intake causes disruption of intestinal integrity and increases germ cell apoptosis. Gliadin-induced germ cell apoptosis (GIGA) was suppressed by depletion of *cep-1*, *ced-13*, *egl-1*, or *mpk-1*. However, HUS-1 was not activated, suggesting that GIGA is activated through the mitogen-activated protein kinase (MAPK) pathway and is CEP-1-dependent but is a separate pathway from that controlling the DNA damage response. Taken together, our results suggest that gliadin causes intestinal barrier disruption through ROS production and interacts with the germ cells to reduce fertility through GIGA.

## 1. Introduction

Wheat is a major food source and is consumed daily worldwide. In recent years, there has been a noticeable increase in the prevalence of gluten-related disorders [1,2]. Many factors have contributed to the increase of gluten-related pathologies, including the worldwide popularization of Western and Mediterranean diets, both of which include high levels of gluten-containing foods [3]. In addition, there is an increasing number of patients with intestinal symptoms related to gluten intake without evidence of celiac disease or wheat allergy, arising from non-celiac gluten sensitivity [4]. Gluten is a complex molecule composed of gliadin and glutenin, and undigested gliadin is known to be a major factor causing gluten toxicity [5]. There are various toxic epitopes in gluten peptides that possess cytotoxic, immunomodulatory, and gut-permeating activities [6], and these have been partially mapped to specific domains in α-gliadin [7]. For example, α-gliadin amino acids 31–43 create a cytotoxic epitope [8,9], residues 57–89 form an immunomodulatory epitope [10,11], residues 111–130 and 151–170 form gut-permeating epitopes [12], and residues 261–277 trigger interleukin (IL)-8 release [13]. Gluten intake is also known to cause disruption of intestinal integrity in humans [14].

In our previous study, we showed that gliadin intake causes the production of reactive oxygen species (ROS) in the intestine of *Caenorhabditis elegans* [15]. ROS are produced in biological systems to modulate cellular activities like cell survival, stress responses, and inflammation [16,17]. An increase in ROS levels is associated with the onset and progression of stress responses. Due to their reactivity, high concentrations of ROS can disrupt the balance between antioxidants and oxidants [17], triggering cellular stress responses, potentially including cell death. We thus hypothesized that gliadin intake by *C. elegans* adults induces intestinal disruption through oxidative stresses, reducing fertility via cell death. *C. elegans* is a powerful model organism to study the biological effects of nutrients, at both the organismal and molecular levels. Additionally, *C. elegans* has many advantages, including highly conserved metabolic pathways, a short life cycle, invariant cell lineages, and well-defined organ development and structure [18,19,20]. To examine whether oxidative stress induced by gliadin intake disrupts intestinal integrity and reduces reproduction at the molecular level, we investigated the effects of gliadin intake on intestinal integrity and germ cell apoptosis in *C. elegans*.

## 2. Materials and Methods

### 2.1. C. Elegans Strains and Gliadin Treatment

*C. elegans* strains were maintained at either 15 or 20 °C on nematode growth medium (NGM) agar plates seeded with *Escherichia coli* strain OP50, as described previously [21]. The following strains were used in this study: N2 (*C. elegans* wild isolate, Bristol variety), CL2166: *dvIs19 ((pAF15)gst-4p::GFP::NLS) III*, CY573: *bvIs5 (cyp-35B1p::GFP+gcy-7p::GFP)*, TK22: *mev-1(kn1) III*, MT1743*: ced-3(n718) IV*, MT2551: *ced-4(n1162) dpy-17(e164) III*, TJ1: *cep-1(gk138) I*, FX536: *ced-13(tm536) X*, MT1082: *egl-1(n487) V*, TG12: *cep-1(lg12501) I; unc-119(ed4) III; gtIs1(CEP-1::GFP+unc-119(+))*, WS1433: *hus-1(op241) I; unc-119(ed3) III; opIs34(hus-1p::hus-1::GFP+unc-119(+))*, AH102: *lip-1(zh15) IV*, SD939: *mpk-1(ga111) unc-79(e1068) III*, NL2098: *rrf-1(pk1417) I*, and NL3511: *ppw-1(pk1425) I*. To examine the effects of gliadin intake, 30 µM gliadin (Sigma-Aldrich, St. Louis, MO, USA) dissolved in DMSO were used based on a previous study [15]. Synchronized L4-stage larvae were treated with gliadin for 24 h at 20 °C and then the adult-stage worms were examined. 

### 2.2. Live Image Observation of Fluorescence-Tagged Transgenic Worms

To observe the expression of glutathione S-transferase 4 (GST-4) and cytochrome P450 oxidase (CYP-35) by gliadin treatment, transgenic strains CL2166 tagged with GFP reporter to the *gst-4* gene (a general indicator of oxidative stress responses) and CY573 tagged with GFP reporter to *cyp-35B1* (a reporter of oxidase detoxification) were used. The synchronized L4-stage of worms expressing GFP were treated with gliadin for 24 h at 20 °C. The worms were then mounted into 0.2 mM tetramisole hydrochloride (Sigma-Aldrich, St. Louis, MO, USA) in M9 buffer on a poly-L-lysine (Sigma-Aldrich, St. Louis, MO, USA) coated glass slide. Live images of worms were observed under a fluorescence microscope (Zeiss Axioscope, Germany).

### 2.3. Treatment with Synthetic Gliadin Peptides and Wheat Gluten Hydrolysate (WGH)

Synthetic gliadin peptides consisting of either aa 31–43, aa 111–130, or aa 151–170 and labelled with either FITC or TAMRA were synthesized by ANYGEN in GwangJu, Korea and used at a final concentration of 3 µM. WGH is a commercial product obtained from Nisshin Pharma (Tokyo, Japan) [22]. A stock WGH solution at 500 mg/mL was prepared in water and WGH was added to NGM plates at a final concentration of 0.5 mg/mL [22]. Wild-type N2 worms were maintained on NGM plates seeded with OP50 containing either 3 µM of synthetic gliadin peptides or 0.5 mg/mL of WGH at 20 °C. Synchronized L4-stage larvae were treated with synthetic gliadin peptides and WGH for 24 h at 20 °C, and then the adult-stage worms were examined. 

### 2.4. Reactive Oxygen Species (ROS) Measurements

To test whether gliadin treatment increases ROS levels in *C. elegans*, ROS production was measured as described previously [15,23]. The animals were transferred to 1 mL of M9 buffer containing 1 µM of 2′,7′-dichlorodihydrofluorescein diacetate (DCFDA) (Sigma-Aldrich, St. Louis, MO, USA). DCFDA is a fluorescent indicator dye that is sensitive to ROS [24]. After 3 h of staining at 20 °C, worms were mounted on 2% agar pads and the DCFDA signal was observed using a fluorescence microscope (Zeiss Axioscope, Germany) with 488 nm excitation and 510 nm emission wave-lengths. The signal was quantified using the Image J software.

### 2.5. Phalloidin Staining

Intestine dissection and phalloidin staining were performed as previously described [25], with minor modifications. To examine intestinal actin, tetramethyl-rhodamine B isothiocyanate (TRITC)-phalloidin (Sigma-Aldrich, St. Louis, MO, USA) was applied. Dissected intestines were fixed with cold methanol and acetone and stained with TRITC-phalloidin for 1 h at 20 °C, then counterstained with 1 µM TO-PRO-3 (Molecular Probes, Eugene, OR, USA) to stain DNA. Samples were imaged under a confocal microscope (Olympus, FV1000 Spectral, Japan) using excitation wave-lengths of 540–545 nm and emission wave-lengths of 570–573 nm, and signals were quantified using Image J.

### 2.6. Intestinal Barrier Function Assay 

Intestinal barrier function assays were performed as previously described [26]. Worms were removed from either NGM or NGM with gliadin plates and suspended for 3 h in liquid cultures of standard OP50 bacteria mixed with blue food dye (FD&C Blue No.1 FD110, Spectrum, New Brunswick, NJ, USA). Worms were then recovered on new OP50-seeded NGM plates and analyzed for the presence or absence of blue food dye in the body cavity using a Zeiss microscope at 40× magnification. For each time point, three independent experiments were performed.

### 2.7. N-Acetyl-L-Cysteine (NAC) Treatment

To examine the impact of antioxidants on effects of gliadin treatment, NAC (Sigma-Aldrich, St. Louis, MO, USA) was used as previously described [15]. Synchronized L4-staged worms were first placed on NGM plates containing 5 mM NAC with either gliadin or synthetic gliadin peptides for 24 h at 20 °C, and then used for further experimentation.

### 2.8. Measurement of Brood Size 

Brood size was calculated as the total number of non-hatched and hatched embryos produced by a single mother hermaphrodite as previously described [15]. We measured brood size of a mother hermaphrodite either treated or non-treated with gliadin.

### 2.9. Germ Cell Apoptosis Assay

Apoptotic germ cells were visualized by acridine orange (AO) vital staining, as previously described [27,28]. Briefly, treated worms were stained with 25 µg/mL AO in M9 buffer for 1 h in the dark, allowed to recover on new NGM plates seeded with OP50 for 20 min in the dark, and the number of AO-positive germ cells per gonad arm was counted under a fluorescence microscope.

### 2.10. RNA Interference (RNAi) Assays

RNAi assays were performed using the soaking method as described previously [28]. dsRNA for *cep-1* and *mpk-1* genes was synthesized in vitro from respective cDNA templates. The cDNA templates flanked by T7 promoter sequences were generated by PCR using the T7 primer, 5′-GTAATACGACTCACTATAGGGC-3′ and the CMo422 primer, 5′-GCGTAATACGACTCACTATAGGGAACAAAAGCTGGAGCT-3′. Soaking buffer without dsRNA was used as the negative mock RNAi control. L1-stage worms were soaked in dsRNA solution for 24 h, then transferred to OP50-seeded NGM plates to be grown for a few days until the worms reached the L4 stage. Worms were then either treated or not treated with gliadin, incubated for an additional 24 h, and the adult-stage worms were examined using the germ cell apoptosis assay.

### 2.11. Immunofluorescence Analysis

Immunofluorescence analysis was performed as previously described [28]. In brief, worms were dissected to extrude gonads in 10 µL of M9 buffer containing 100 µg/mL tetramisole on a poly-L-lysine-coated slide, covered with a coverslip, freeze-cracked with liquid nitrogen, fixed with cold methanol and acetone, and immunostained with primary and secondary antibodies. The specimens were further counterstained with 1 µM TO-PRO-3 (Molecular Probes) to stain DNA and observed under a confocal microscope (Olympus, FV1000 Spectral, Tokyo, Japan). The following antibodies were used: rabbit anti-GFP (1:400; Novus, USA), rabbit anti-p44/42 MAPK (1:400; Cell signaling technology, Danvers, MA, USA), and anti-rabbit IgG (Alexa Fluor 488 conjugated) (1:500; Invitrogen, Carlsbad, CA, USA).

### 2.12. Cell Culture and Cell Viability Assays

Murine RAW 264.7 macrophage cells were obtained from the American Type Culture Collection (ATCC, Manassas, VA, USA). Cells were cultured in Dulbecco’s modified Eagle’s medium (DMEM with high glucose, HyClone, Logan, UT, USA) with 10% fetal bovine serum (HyClone) and 1% penicillin–streptomycin (HyClone) in a humidified incubator at 37 °C under a 5% CO_2_ atmosphere. The cytotoxicity of synthetic gliadin peptides was measured using the 3-(4,5-dimethylthiazole-2-yl)-2,5-diphenyltetrazolium bromide (MTT; Sigma-Aldrich, St. Louis, MO, USA) assay as follows. Cells were cultured in 96-well plates containing 1 × 10^4^ cells/well in media for 24 h. The medium in each well was exchanged for fresh DMEM containing various concentrations (0.1 µM, 0.25 µM, 0.5 µM, 1 µM, 1.5 µM, or 3 µM) of gliadin peptide (GP): GP31-43, GP111-130, or GP151-170. After 24 h of incubation, the cells were further incubated with MTT for 4 h. The medium was then removed and the formazan precipitate was solubilized in DMSO. The optical density of each well was then determined at 570 nm using a microplate reader (TECAN, Mannedorf, Switzerland).

### 2.13. Intracellular ROS Accumulation Measurements

To measure intracellular ROS, RAW 264.7 macrophages were seeded in a 6-well plate at 1 × 10^6^ cells/well. After 24 h of incubation, the cells were treated with GP151-170 for 2 h and, then, stimulated with 1 µg/mL lipopolysaccharide (LPS; Sigma-Aldrich, St. Louis, MO, USA) for 24 h. Cells were washed using cold phosphate buffered saline (PBS; Gibco BRL, Grand Island, NY, USA) three times, and then stained for 30 min with 20 µM 2′,7′-dichlorofluorescin diacetate (DCFDA; Sigma-Aldrich, St. Louis, MO, USA) dissolved in serum-free DMEM. DCFDA fluorescence was observed under an inverted fluorescence microscope (Olympus IX70, Okaya, Japan). Fluorescence intensity was quantified using a microplate reader with excitation at 488 nm and emission at 538 nm.

### 2.14. Statistical Analysis

All experiments were repeated more than three times for statistical evaluation of the data. The two-tailed Student’s *t*-test was used to calculate *p* values and *p* < 0.05 was considered significant. The in vitro data were analyzed using the Prism 8 software (GraphPad Software, San Diego, CA, USA). The data are expressed as the mean ± standard deviation (SD). Quantitative data were compared among groups by one-way ANOVA. When the ANOVA results indicated significance (*p* < 0.05), Dunnett’s multiple comparison tests were performed.

## 3. Results

### 3.1. Gliadin Intake Induces GST-4, CYP-35 and ROS Production in Adult-Stage C. elegans 

**Figure 1:**
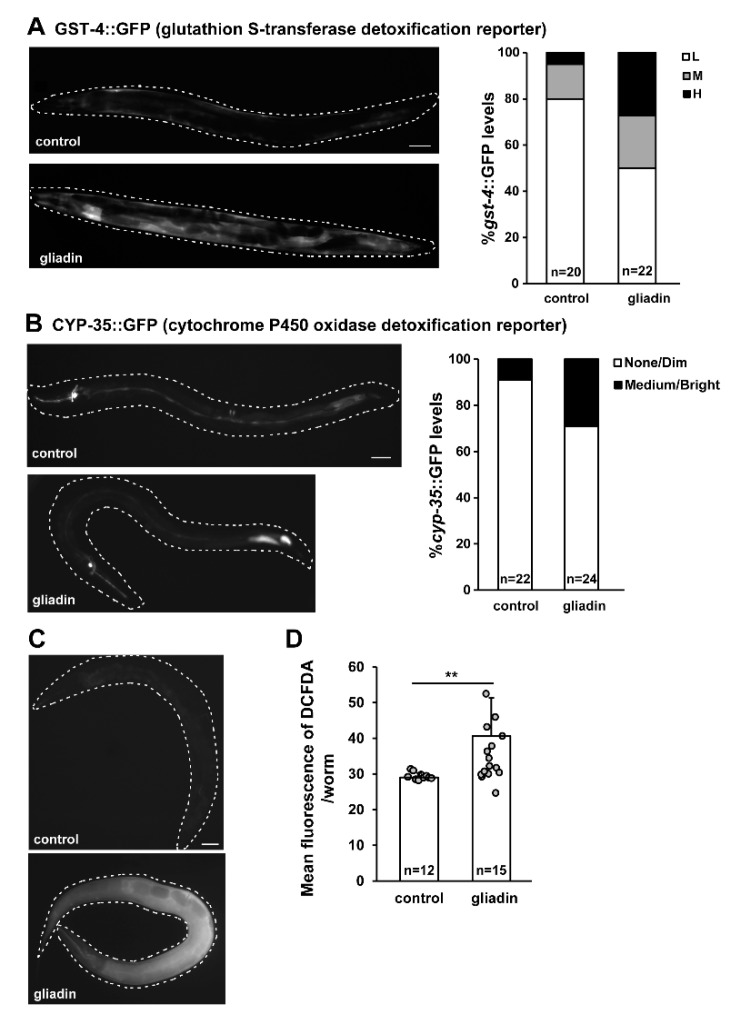
Gliadin intake-induced glutathione S-transferase 4 (GST-4), cytochrome P450 oxidase (CYP-35), and reactive oxygen species (ROS) production in adult-stage *Caenorhabditis elegans* worms. (**A**) *Pgst-4::GFP* transgenic populations synchronized at the L4 larval stage were treated with gliadin for 24 h at 20 °C. Pictures show representative images of *Pgst-4::GFP* expression after 24 h of gliadin treatment. The bar graph (right panel) shows quantified induction levels of the *Pgst-4::GFP* reporter in the intestine categorized as low (L), medium (M), and high (H) based on a previously established scale [31]. Scale bar, 100 μm. (**B**) *cyp-35::GFP* transgenic populations synchronized at the L4 larval stage were treated with gliadin for 24 h at 20 °C. Pictures show representative images of *cyp-35::GFP* expression after 24 h of gliadin treatment. The bar graph shows the distribution of GFP expression levels after gliadin treatments. Scale bar, 100 μm. (**C**) Wild-type N2 animal populations synchronized at the L4 larval stage were treated with gliadin or left untreated as controls. ROS production was measured by 2′,7′-dichlorodihydrofluorescein diacetate (DCFDA) staining after 24 h of treatment. Pictures show representative images of ROS production after treatment as visualized by DCFDA staining (left panels). (**D**) Bar graph showing pixel intensities from DCFDA fluorescence per worm. Error bars represent s.d. **p < 0.005 (Student’s *t*-test).

We previously showed that gliadin induces oxidative stress responses and reactive oxygen species (ROS) production in the early larval stage of *C. elegans* [15]. In this study, we examined the effects of gliadin on adult-stage *C. elegans* in which major developmental processes have been completed, allowing for observation of physiological effects of gliadin without interference from developmental processes. We first examined whether gliadin intake induces the same stress responses in the adult stage as in the larval stage by measuring the expression levels of glutathione S-transferase 4 (*gst-4*, a general indicator of oxidative stress responses [29,30]), and cytochrome P450 oxidase (*cyp-35*, a reporter of detoxification). Adult-stage transgenic worms expressing either GST-4::GFP or CYP-35::GFP were examined after gliadin intake and showed higher expression levels of both GST-4::GFP and CYP-35::GFP (Figure 1A,B). This indicates that GST-4 and CYP-35 were indeed induced upon gliadin intake in the adult-stage *C. elegans* worms. Higher levels of ROS production in gliadin-treated worms were observed by treatment with 2′,7′-dichlorodihydrofluorescein diacetate (DCFDA), a dye that is cleaved intracellularly and fluoresces green upon exposure to intracellular ROS (Figure 1C). Taken together, these experiments indicate that gliadin intake induces GST-4, CYP-35 and ROS production in the adult *C. elegans*.

### 3.2. ROS Production Is Induced by the Intake of Synthetic Gliadin Peptides but not Wheat Gluten Hydrolysate (WGH) in Adult-Stage C. elegans

**Figure 2:**
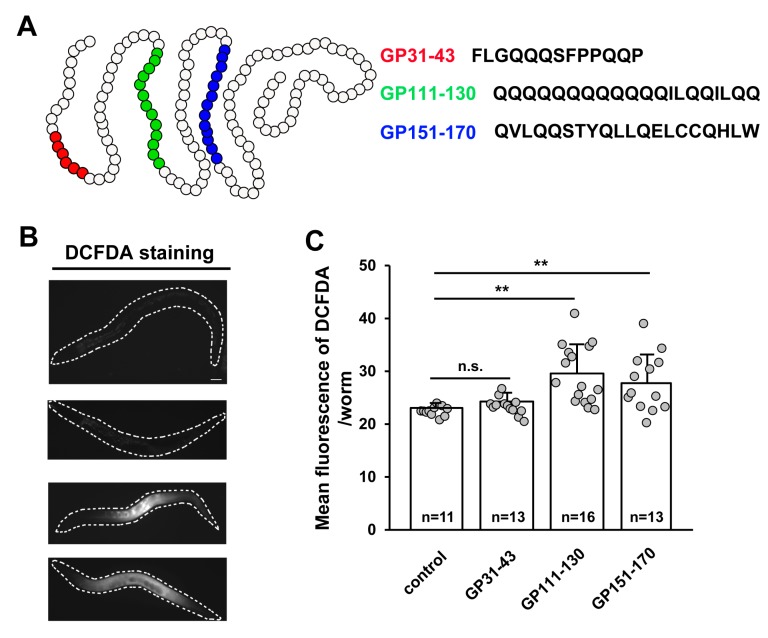

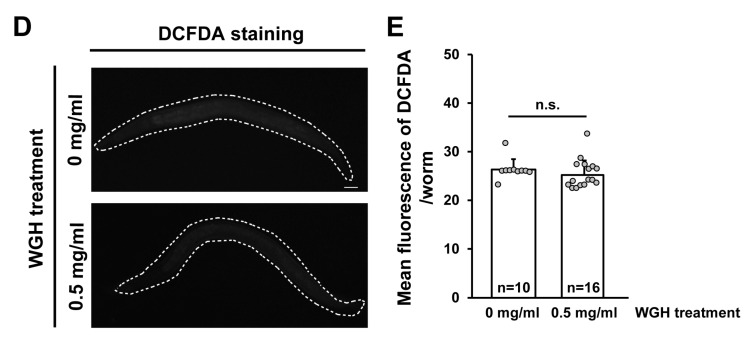
Effects of treatment with synthetic gliadin peptides or wheat gluten hydrolysate (WGH) on reactive oxygen species (ROS) production in adult-stage *C. elegans* worms. (**A**) Schematic of α-gliadin motifs with respective peptide sequences. Three kinds of synthetic α-gliadin peptides were generated that either possess cytotoxic activity (red) or gut-permeating activity (green or blue) as previously reported [7]. (**B**,**C**) Wild-type N2 animal populations synchronized at the L4 larval stage were treated with synthetic gliadin peptides and ROS production was measured by 2′,7′-dichlorodihydrofluorescein diacetate (DCFDA) staining 24 h after treatment. Pictures show representative images of DCFDA staining indicating ROS production after treatment (**B**). Bar graph (**C**) shows the pixel intensities from DCFDA fluorescence per worm. Error bars represent s.d.; n.s., not significant; ** *p* < 0.005 (Student’s *t*-test). (**D**,**E**) Wild-type N2 animal populations synchronized at the L4 larval stage were treated with WGH and ROS production measured by DCFDA staining after 24 h of treatment. Pictures show representative images by DCFDA staining indicating ROS production after treatment (**D**). Bar graph (**E**) shows the pixel intensities from DCFDA fluorescence per worm. Error bars represent s.d. n.s., not significant. (Student’s *t*-test).

There are three motifs identified in α-gliadin that possess cytotoxic, immunomodulatory, or gut-permeating activity in vitro [6,7]. We also attempted to confirm the intake of gliadin by feeding worms with labeled synthetic gliadin peptides for visualization. The labeled synthetic peptides were found in the intestine of worms, indicating that worms were able to consume gliadin. Because *C. elegans* does not have an adaptive immune system, cytotoxic and gut-permeating motifs but not immunomodulatory motifs tested their effects. These peptides represent different portions of gliadin (Figure 2A); that is, a cytotoxic peptide from aa 31 to 43 (GP31–43) [8,9,32] and two gut-permeating peptides from aa 111 to 130 (GP111–130) and from aa 151 to 170 (GP151–170) [12]. Based on previous reports [8,9,12,32], we hypothesized that treating adult *C. elegans* worms with these α-gliadin motifs may have similar effects to gliadin treatment. All three synthetic peptides caused increases in ROS production; however, such increases were only statistically significant after treatment with GP111–130 and GP151–170 (Figure 2B,C). These results suggest that the intake of GP111–130 and GP151–170, which are known gut-permeating peptides, increases ROS production in *C. elegans*. It has been reported that wheat gluten hydrolysate (WGH) shows beneficial effects on *C. elegans* [22]; therefore, we examined the effects of WGH intake on ROS production in adult-stage *C. elegans*. We measured ROS production after WGH treatment and found that WGH treatment did not induce ROS production (Figure 2D,E); this suggests that ROS production is a gliadin-specific response. 

**Figure 3:**
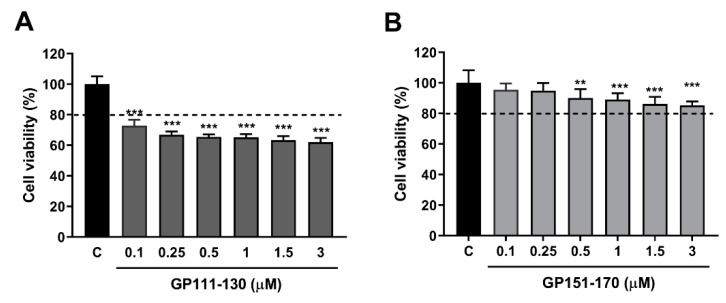

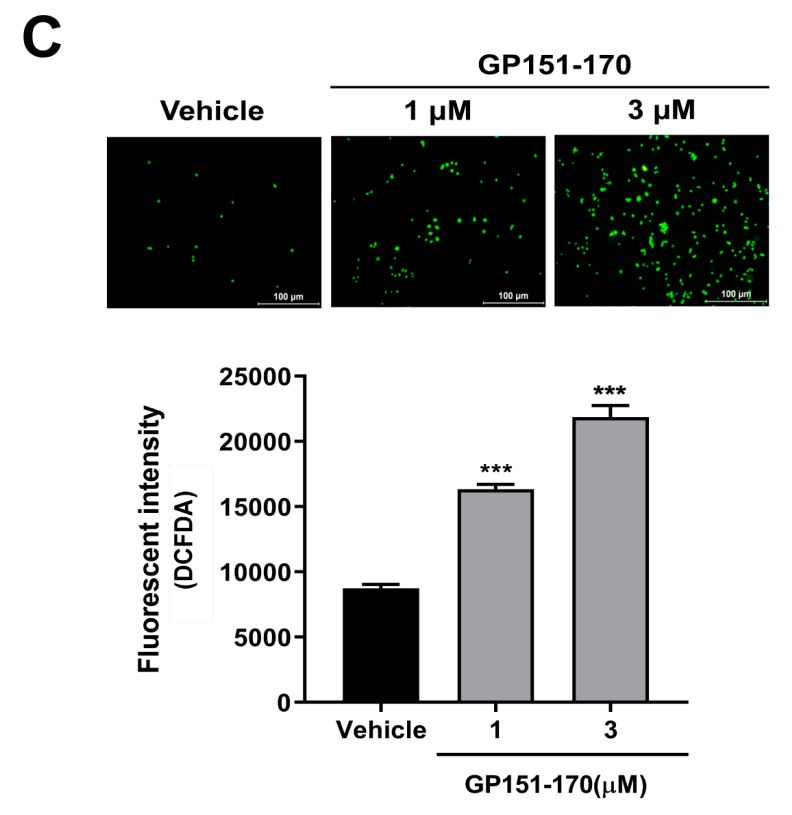
Effects of the synthetic gliadin peptide GP151–170 on reactive oxygen species (ROS) production in mammalian cell cultures. (**A**,**B**) Cell viability in RAW264.7 macrophages after treatment with either the synthetic gliadin peptide GP110–130 or GP151–170. (**C**) Intracellular ROS levels were evaluated by measuring the intensity of 2′,7′-dichlorodihydrofluorescein diacetate (DCFDA) fluorescence using a microplate reader. A bar graph showing the pixel intensities of DCFDA fluorescence is shown. Error bars represent s.d. ** *p* < 0.01. *** *p* < 0.001 (Student’s *t*-test). C, control.

We further investigated the oxidative stress response to gliadin in mammalian cells using synthetic peptides. Among three synthetic gliadin peptides, GP31–34 showed cell lethality (data not shown) and GP111–130 treatment resulted in 60% cell viability (Figure 3A). RAW264.7 macrophages treated with either 1 µM or 3 µM GP151–170 showed 89.04 ± 1.60% and 85.21 ± 1.00% cell viability, respectively, compared to control cells (Figure 3B). We therefore examined ROS production in the RAW264.7 macrophages treated with the GP151–170 synthetic peptide. Cells were treated with either 1 µM or 3 µM of GP151–170, and ROS production was measured by DCFDA staining. Treatment with GP151–170 caused a high induction of ROS production compared to that in non-treated control cells (Figure 3C). These results indicate that gliadin intake triggers oxidative stress responses through ROS production.

### 3.3. Intestinal Integrity is Disrupted by Gliadin Intake 

**Figure 4:**
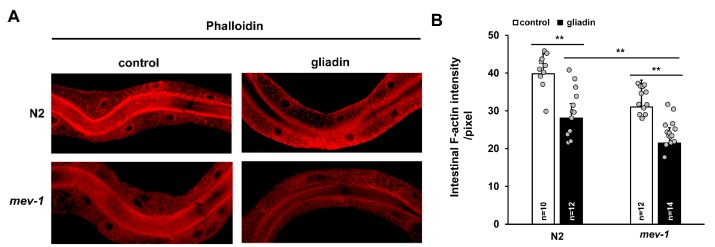
Effects of gliadin treatment on intestinal F-actin in adult-stage wild-type N2 and *mev-1* mutant *C. elegans* worms. (**A**,**B**) Wild-type N2 and *mev-1* mutants synchronized at the L4 larval stage were treated with or without gliadin. Detection of phalloidin staining of intestinal F-actin reveals a significant reduction in fluorescence after gliadin treatment in either N2 and *mev-1* mutants based on quantification by image J analysis (**B**). Error bars represent s.d. ** *p* < 0.005 (Student’s *t*-test).

ROS play roles in pathogenesis during several intestinal disorders, including inflammatory bowel disease and necrotizing enterocolitis [33,34,35], and cause disruption of intestinal tight junctions and barrier function in mammals [36]. Based on these findings, we investigated the effects of gliadin intake on intestinal epithelial cells in *C. elegans*. Because F-actin is apically enriched in *C. elegans* intestine and accumulates at the apical surface of the gut [37], we analyzed intestinal F-actin intensity using phalloidin staining after gliadin intake. We found that intestinal F-actin levels decrease upon gliadin intake (Figure 4A,B) and that this gliadin-induced decrease is more severe in oxidative stress-sensitive *mev-1* mutants compared to that in wild-type N2 worms (Figure 4A,B).

**Figure 5:**
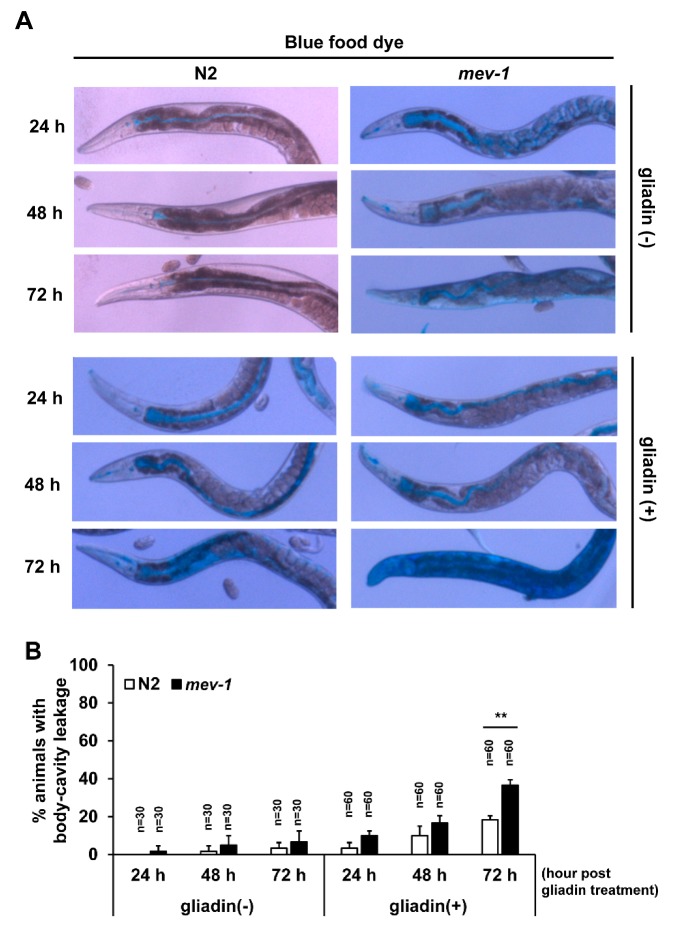
Effects of gliadin treatment and age on intestinal barrier function in wild-type N2 and *mev-1* mutant *C. elegans* worms. (**A**,**B**) Wild-type N2 and *mev-1* mutants synchronized at the L4 larval stage were treated with or without gliadin then soaked in blue food dye for 3 h on respective hours of gliadin treatment at adulthood. Differential interference contrast (DIC) images of wild-type N2 and *mev-1* mutants (**A**) after soaking in blue food dye for 3 h after the indicated hours of gliadin treatment. Quantification of body-cavity leakage in wild-type N2 and *mev-1* mutants (**B**) after indicated lengths of gliadin treatment during adulthood. Error bars represent s.d. ** *p* < 0.005 (Student’s *t*-test).

We further assessed a possible link between the loss of F-actin and intestinal integrity by examining the leakiness of intestinal epithelial cells using blue food dye in both wild-type N2 and the *mev-1* mutant, as previously described [26]. The dye was visible in the intestines of non-treated wild-type and *mev-1* mutant worms, and gliadin-treated wild-type and *mev-1* mutant worms, suggesting that dye was equally consumed by these four groups of worms (Figure 5A). In non-treated wild-type N2 worms, dye leakage was first observed after 48 h incubation, while it was observed in worms after 24 h after gliadin treatment. Similarly, gliadin treatment enhanced level of dye leakage in *mev-1* mutants. Furthermore, dye leakage was observed in around 20% of *mev-1* mutant worms at 48 h after gliadin treatment and dramatically increased with time to include 40% of *mev-1* mutant worms at 72 h after gliadin treatment (Figure 5A,B). These results suggest that gliadin treatment causes disruption of intestinal integrity and that this disruption is enhanced with age.

### 3.4. Effects of Gliadin and Synthetic Gliadin Peptides on Intestinal F-Actin Formation Are Suppressed by Antioxidant Treatment

**Figure 6:**
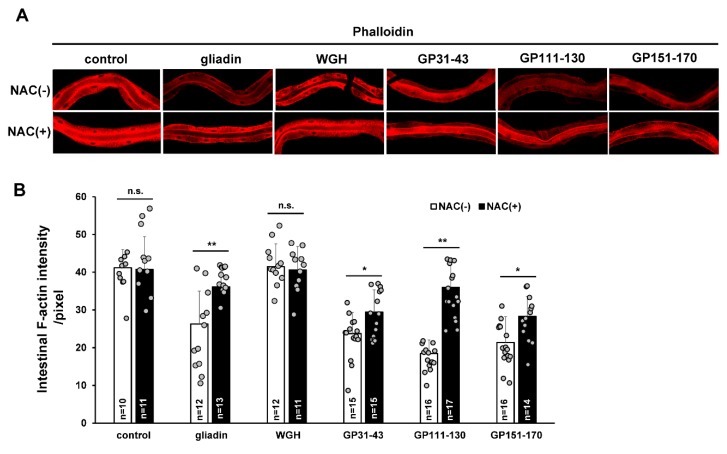
Effects of combining N-acetyl-L-cysteine (NAC) treatment with treatment by gliadin or synthetic gliadin peptides on intestinal F-actin in adult-stage wild-type N2 *C. elegans*. (**A**,**B**) Wild-type N2 worms synchronized at the L4 larval stage were treated with or without NAC and either no fed (control), fed gliadin, wheat gluten hydrolysate (WGH), or synthetic gliadin peptides (GP31–43, GP111–130, or GP151–170) for 24 h. Detection of intestinal F-actin by phalloidin staining reveals a significant increase in fluorescence (quantification by image J analysis in (**B**)) in worms treated with both NAC and either gliadin, GP111–130, or GP151–170). Error bars represent s.d. n.s., not significant. * *p* < 0.05. ** *p* < 0.005 (Student’s *t*-test).

Disrupted intestinal tight junctions and the cytoskeleton via an oxidative stress-dependent mechanism has been reported to be inhibited by antioxidant feeding in a mouse model [38]. To determine whether the reduction of intestinal F-actin levels by gliadin-induced oxidative stress can be similarly repressed by antioxidants, we treated control-, WGH-, and gliadin-fed worms with the antioxidant N-acetyl-L-cysteine (NAC). We found that NAC treatment significantly suppressed the gliadin-triggered reduction in intestinal F-actin levels (Figure 6A,B). We also treated worms with the labelled synthetic gliadin peptides to see if specific regions of gliadin are involved in the disruption of intestinal integrity in worms. We assumed that these peptides were taken up by the intestine in equal amounts. Intestinal F-actin intensity was reduced by treatment with each synthetic gliadin peptide, and this reduction was significantly suppressed by NAC treatment (Figure 6A,B). These observations confirm that ROS production induced by gliadin intake reduces intestinal F-actin formation.

### 3.5. Gliadin Intake Increases Germ Cell Apoptosis

**Figure 7:**
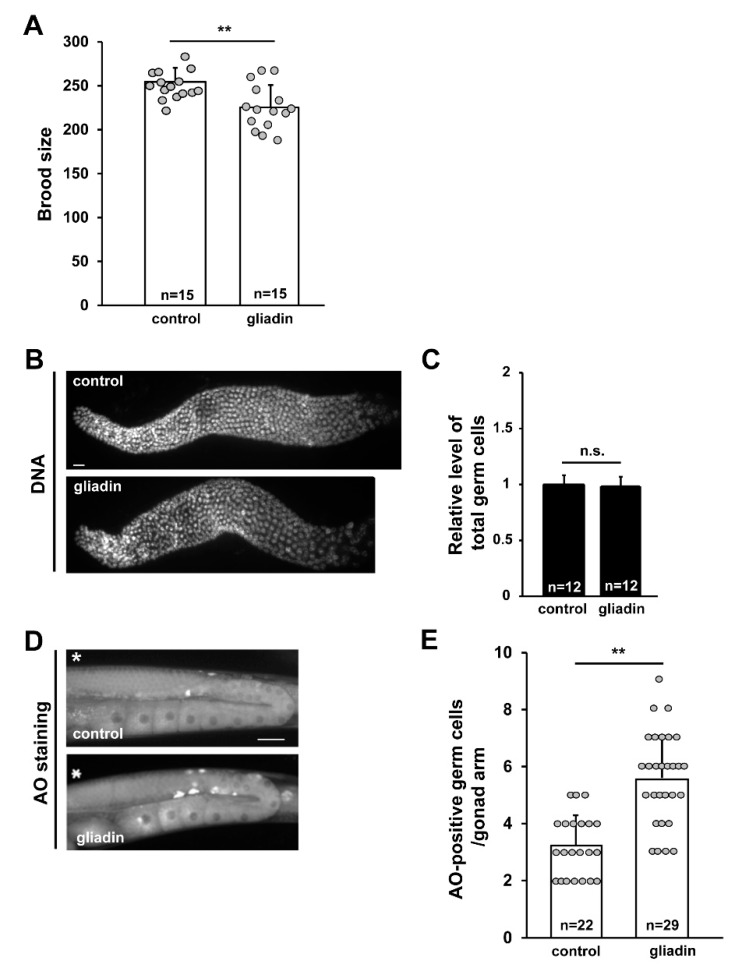
Effect of gliadin intake on germ cell proliferation and apoptosis in adult wild-type N2 *C. elegans* worms. (**A**) The brood size of gliadin-treated mothers compared to control mothers after 4 days. ** *p* < 0.005. (**B**,**C**) Germ cell proliferation was not affected by gliadin treatment. Wild-type N2 worms synchronized at the L4 larval stage were treated with or without gliadin for 24 h, and dissected gonads were DNA-stained using TO-PRO-3. Bar graph (**C**) shows relative levels of germ cell proliferation of gliadin-treated worms compared to control worms. (**D**,**E**) Germ cell apoptosis increases with gliadin treatment as indicated by staining apoptotic germ cells with acridine orange (AO). *, Distal end of each gonad arm. Bar, 20 μm. ** *p* < 0.005 (Student’s *t*-test).

**Figure 8:**
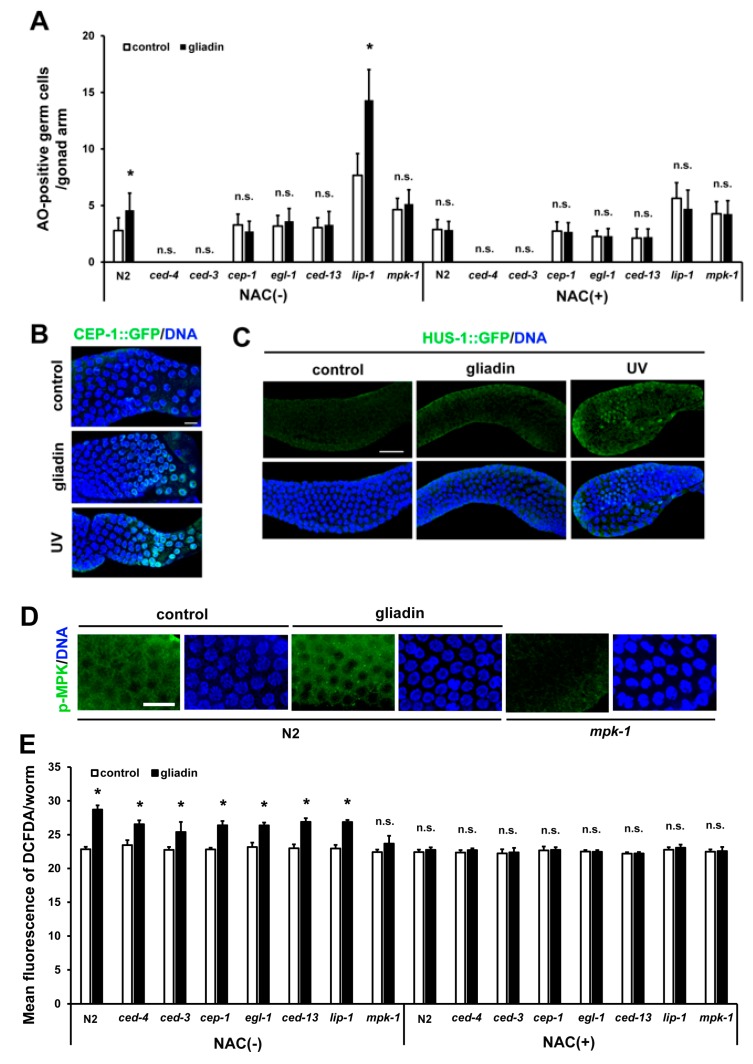
Effects of CEP-1 activity and mitogen-activated protein kinase (MAPK) signaling on gliadin-induced germ cell apoptosis (GIGA) in adult *C. elegans* worms. (**A**) Average numbers of acridine orange (AO)-positive germ cells per gonad arm in wild-type N2, *ced-4*, *ced-3, cep-1, egl-1, ced-13, lip-1*, and *mpk-1* mutants (*n* = 30–40 per group) after gliadin treatment for 24 h of treatment with N-acetyl-L-cysteine (NAC) (+) or without NAC (–) starting at the L4 larval stage. (**B**) CEP-1::GFP was observed by immunostaining in CEP-1::GFP transgenic animals (*n* = 30 per group) fed with or without gliadin for 24 h starting in the L4 larval stage. UV-treatment condition was used as a positive control. Bar, 10 μm. (**C**) HUS-1::GFP transgenic animals (*n* = 30 per group) were synchronized at the L4 stage then treated with or without gliadin. HUS-1::GFP aggregates were observed after UV-treatment (positive control) by immunostaining using an anti-GFP antibody. Bar, 20 μm. (**D**) Phospho-MPK was observed by immunostaining using an anti-Phospho-p44/42 MAPK antibody in wild-type N2 worms (*n* = 32 per group) after gliadin treatment for 24 h starting during the L4 larval stage. Bar, 10 μm. (**E**) Wild-type N2, *ced-4*, *ced-3, cep-1, egl-1, ced-13, lip-1*, and *mpk-1* mutants (*n* = 30–40 per group) after gliadin treatment for 24 h treated with NAC (+) or without NAC (–) starting at the L4 larval stage. Reactive oxygen species (ROS) production was measured by 2′,7′-dichlorodihydrofluorescein diacetate (DCFDA) staining. The bar graph shows the pixel intensities from DCFDA fluorescence per worm. Error bars represent s.d.; n.s., not significant. * *p* < 0.05 (Student’s *t*-test).

Anatomically, the intestine and the germ cells are very closely located in *C. elegans*. The close link between nutrient export from the intestine and the production of gametes by the germ line was previously proposed [39]. Additionally, the existence of a crosstalk between the intestine and the germ line has been shown through the removal of germline stem cells, which resulted in increased fat accumulation in the intestine derived from unconsumed yolk [40,41]. In addition, our previous study showed that gliadin intake reduces reproduction in *C. elegans* [15]. To examine how the gliadin intake reduces fertility and whether intestinal changes can affect reproduction in the gonads, we further investigated the effect of gliadin intake on the germ line in adult-stage *C. elegans*. We confirmed the reduction in brood size after gliadin intake by L4 stage worms (Figure 7A). We next asked whether this reduced brood size is caused by a lower total number of germ cells or an increase in the level of apoptotic germ cells. In adult worms, after gliadin intake there was no significant change in the total number of germ cells (Figure 7B,C), while there was a significant increase in the level of germ cell apoptosis (Figure 7D,E). We further investigated gliadin-induced germ cell apoptosis (GIGA) in *ced-3* and *ced-4 C. elegans* mutants, which have mutations in core apoptotic machinery genes. CED-3 is a caspase-family protease (Caspase-3) and CED-4 is a mammalian apoptotic protease-activating factor-1 (Apaf-1). As shown in Figure 8A, GIGA is abolished in *ced-3* and *ced-4 C. elegans* mutants. 

It has been proposed that the mammalian p53 tumor suppressor protein CEP-1 mediates stress responses in somatic cells and apoptosis in germ cells [42]. Additionally, germ cell apoptosis mediated by the ER stress response sensor IRE-1 requires CEP-1 [43]. Based on these findings, we examined whether CEP-1 activity is required for the increase of germ cell apoptosis triggered by gliadin intake. We found that GIGA is suppressed by the depletion of *cep-1* and its downstream target genes *egl-1* and *ced-13* (Figure 8A), which are *C. elegans* orthologs of a pro-apoptotic BH-3 protein. These results indicate that GIGA is dependent on *cep-1* activity. We also confirmed an increase in CEP-1 levels after gliadin treatment by immunostaining of the germ line of CEP-1::GFP transgenic worms (Figure 8B). Moreover, we examined the possibility of DNA damage in the germ line due to gliadin intake using HUS-1::GFP transgenic worms, which are usually used to examine DNA damage in *C. elegans* [44]. There was no significant difference in HUS-1 activity in the germ line after gliadin treatment (Figure 8C), indicating that GIGA requires CEP-1 activity but is independent of the DNA damage response. 

The mitogen-activated protein kinase (MAPK) signaling pathway is a well-known signal transduction pathway that regulates a variety of cellular activities [45]. MAPK also appears to modulate endosulfan-induced germ cell apoptosis in *C. elegans* [46]. We, therefore, investigated the possible involvement of MAPK in GIGA. We observed an increased level of phospho-MPK in germ cells by immunostaining after gliadin treatment (Figure 8D). In addition, germ cell apoptosis increased upon gliadin intake in wild-type N2 worms but not in *mpk-1* mutants. GIGA was, thus, not induced in *mpk-1* mutants, suggesting that GIGA is dependent on the activity of *mpk-1*, an ortholog of human *MAPK1* and *MAPK3* (Figure 8A). These results suggest that GIGA is activated through the MAPK signaling pathway. In addition, mutations in *lip-1*, a negative regulator of *mpk-1*, led to high increases in GIGA (Figure 8A). This result demonstrates that *lip-1* mutants show hypersensitivity to gliadin treatment. We further asked whether this hypersensitivity in the *lip-1* mutants is possibly suppressed by NAC treatment. We observed that a high level of germ cell apoptosis upon gliadin intake both in wild-type N2 and *lip-1* mutants was significantly suppressed by NAC treatment (Figure 8A). These results suggest that gliadin-induced ROS production increases germ cell apoptosis. We further measured gliadin-induced ROS production in these mutants and found that gliadin-induced ROS production was significantly increased in all mutants examined except *mpk-1* mutant (Figure 8E). The increased ROS production was also suppressed by NAC treatment (Figure 8E).

**Figure 9:**
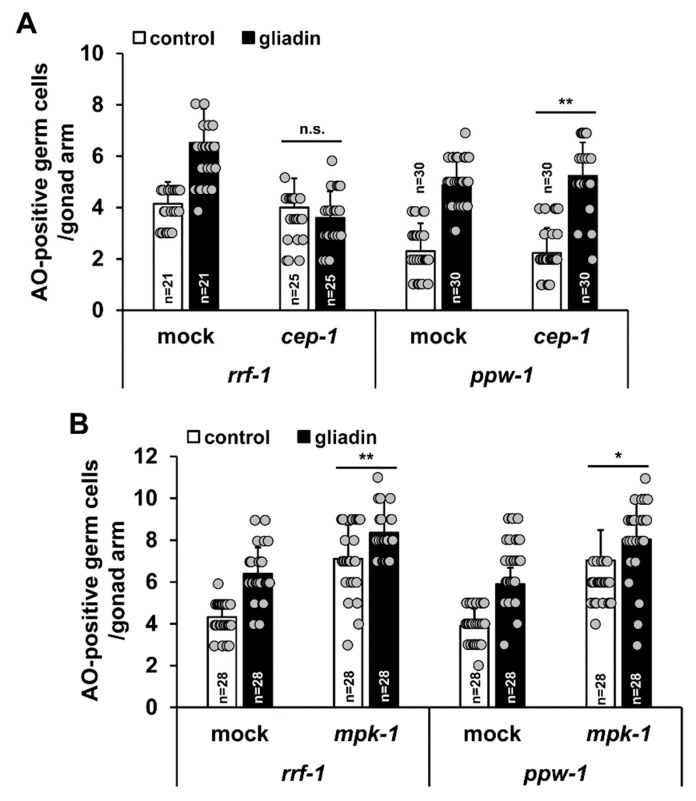
*cep-1* and *mpk-1* function in different tissues is required for increases in germ cell apoptosis triggered by gliadin intake. (**A**,**B**) After RNA interference (RNAi) treatment of control, *cep-1*, or *mpk-1* worms at the L1 stage, worms were grown in nematode growth medium (NGM) plates and treated with or without gliadin for starting at the L4 stage for 24 h. Acridine orange (AO)-positive germ cells were observed in either RNAi-treated *rrf-1* or *ppw-1* mutant backgrounds 24 h after gliadin treatment. n.s., not significant. * *p* < 0.05. ** *p* < 0.005 (Student’s *t*-test).

Next, we asked whether gliadin intake triggers germ cell apoptosis autonomously or non-autonomously. We, thus, used RNAi to deplete *cep-1* and *mpk-1* in *rrf-1* and *ppw-1* mutants. The *rrf-1* and *ppw-1* mutants are used for germline-biased and soma-biased RNAi depletion, respectively [47,48]. *cep-1* depletion by RNAi in *rrf-1* mutants suppressed GIGA, as observed in *cep-1* mutants (Figure 8A and Figure 9A). Conversely, depletion of *cep-1* by RNAi in the *ppw-1* mutants did not suppress GIGA (Figure 9A). These results clearly indicate that *cep-1* is required in the germ line, but not in somatic cells, for normal levels of GIGA. We also observed that *mpk-1* RNAi depletion did not suppress GIGA in both *rrf-1* and *ppw-1* mutant backgrounds (Figure 9B), suggesting that *mpk-1* is required in both somatic and germ cells to promote GIGA. 

### 3.6. Effects of Gliadin and Synthetic Gliadin Peptides on Germ Cell Apoptosis are Suppressed by Antioxidant Treatment

**Figure 10:**
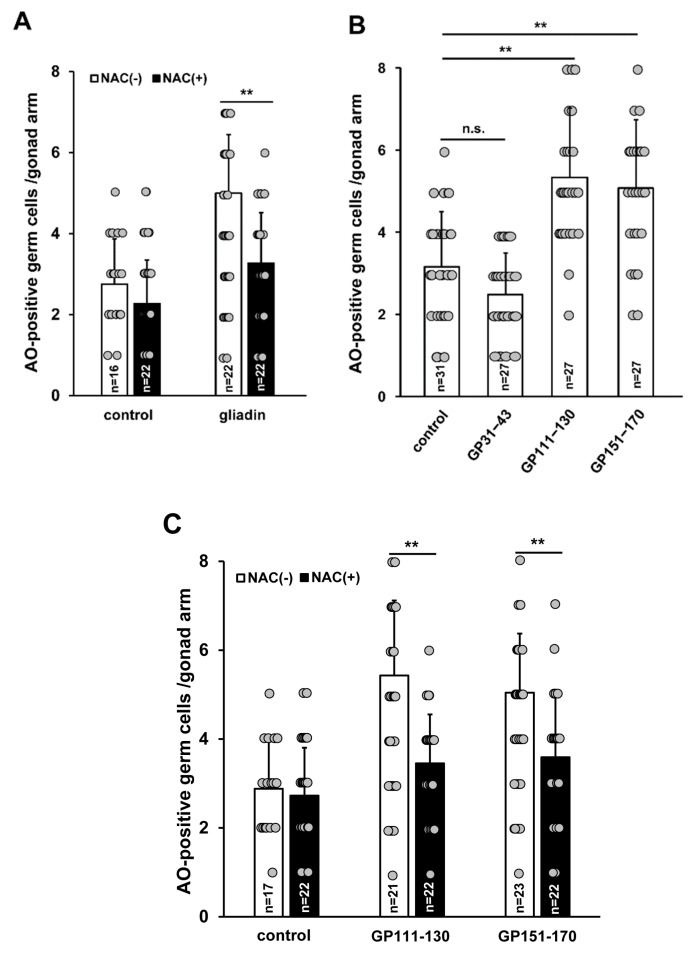
Effect of N-acetyl-L-cysteine (NAC) treatment on germ cell apoptosis triggered by intake of gliadin or synthetic gliadin peptides in adult wild-type N2 *C. elegans* worms. (**A**) Average numbers of acridine orange (AO)-positive germ cells per gonad arm in gliadin-treated wild-type N2 worms fed with or without NAC. (**B**) Average numbers of AO-positive germ cells per gonad arm in wild-type N2 after treatment with synthetic gliadin peptides (GP31–43, GP111–130, or GP151–170). (**C**) Average numbers of AO-positive germ cells per gonad arm in synthetic gliadin peptide (GP111–130 and GP151–170)-treated wild-type N2 worms fed with or without NAC. n.s., not significant. ** *p* < 0.005 (Student’s *t*-test).

We found that antioxidant treatment prevents the reduction in intestinal F-actin intensity triggered by gliadin intake (Figure 6A,B). In addition, we observed that antioxidant treatment can suppress GIGA (Figure 8 and Figure 10A). Since both gliadin-induced ROS and GIGA were suppressed by NAC treatment, we examined whether these changes were reflected in the synthetic gliadin peptides treatment (GP31–43, GP111–130, and GP151–170). Treatment with GP111–130 and GP151–170, but not GP31–43 significantly increased GIGA (Figure 10B), consistent with increases in ROS production triggered by GP111–130 and GP151–170. This suggests that ROS production contributes to an increased level of GIGA. Additionally, to determine the effect of antioxidant treatment in worms fed with GP111–130 and GP151–170, we examined GIGA levels in worms co-treated with a synthetic gliadin peptide and NAC. We observed that GIGA increase after GP111–130 or GP151–170 intake was significantly suppressed by the addition of NAC (Figure 10C), indicating that GIGA is activated by oxidative stress.

### 3.7. Effect of Antioxidants on Gliadin-Induced ROS and Germ Cell Apoptosis in An Oxidative Stress-Sensitive Mutant

**Figure 11:**
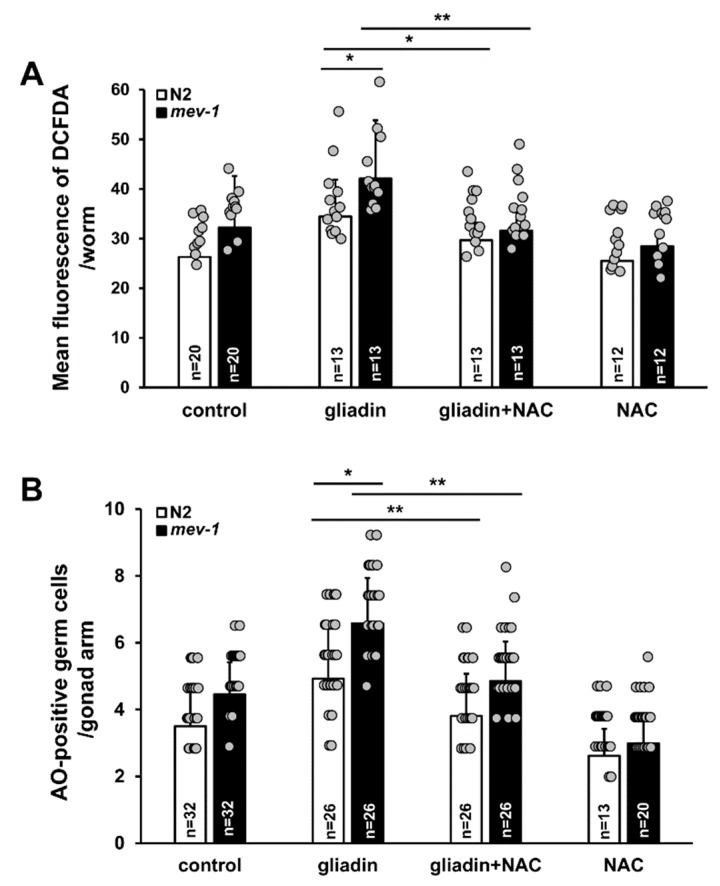
Effects of antioxidants on *mev-1* mutants after intake of either gliadin or a synthetic gliadin peptide. (**A**) Bar graph showing the pixel intensities of 2′,7′-dichlorodihydrofluorescein diacetate (DCFDA) fluorescence per worm for gliadin-treated wild-type N2 and *mev-1* mutant worms fed either with or without N-acetyl-L-cysteine (NAC). (**B**) Average numbers of acridine orange (AO)-positive germ cells per gonad arm in gliadin-treated wild-type N2 and *mev-1* mutant worms fed with or without NAC. Error bars represent s.d. * *p* < 0.05. ** *p* < 0.005 (Student’s *t*-test).

*mev-1* is an ortholog of human succinate dehydrogenase complex subunit C, which regulates oxidative stress responses through its electron transfer activity [49]. The *mev-1* mutant of *C. elegans* is an oxidative stress-sensitive strain. In the previous results, we showed that *mev-1* is hypersensitive to gliadin treatment due to the effects of gliadin treatment on intestinal F-actin formation and intestinal barrier function (Figure 4 and Figure 5). We thus examined gliadin-induced ROS production and GIGA in *mev-1* mutants. Higher levels of ROS production after gliadin intake were observed in *mev-1* mutants compared to those in wild-type N2 worms (Figure 11A). Furthermore, this increase in gliadin-triggered ROS was significantly suppressed by NAC treatment in both N2 and *mev-1* mutants (Figure 11A). We also observed a higher level of GIGA in *mev-1* mutants compared to that in N2 worms (Figure 11B); this level was suppressed by NAC treatment in both N2 and *mev-1* mutants, confirming that antioxidants play critical roles in maintaining normal levels of ROS production and suppressing GIGA. These findings strongly indicate a relationship between ROS production in the intestine and the increase in the level of GIGA in *C. elegans*.

## 4. Discussion

The impact of gluten toxicity on intestinal stress in non-celiac disease patients has been poorly studied; however, it is an important topic for understanding the effects of food consumption in populations with a diet rich in high gluten foods. In a previous study, we developed a simple *C. elegans* animal model system to investigate the effects of gluten toxicity on animal development, behavior, survival, and pathophysiology. We found that gliadin intake induces oxidative-stress responses in the intestine and decreases reproduction [15]. In *C. elegans*, the intestine and the gonad are major organs and are closely located in the body. These two organs, therefore, may communicate with each other more intimately than with any other organs in *C. elegans*. In addition, both organs are anatomically well defined in *C. elegans* [50]. *C. elegans* is, therefore, a good model to study the impact of food-induced intestinal stress on reproduction. Furthermore, the *C. elegans* genome has been sequenced and *C. elegans* contains homologs of 60–80% of the known human genes [51,52,53]. In addition, tools for studying genetics are well established for *C. elegans*, which is advantageous for studying the in vivo functions of genes underlying the molecular mechanisms of food intake and resulting effects on the intestine and the germ cells. Here, we investigated the effects of a major wheat gluten component, gliadin, on the *C. elegans* model and found that gliadin intake induces ROS production in the intestine, disrupts the intestinal integrity, and induces a high level of germ cell apoptosis in the gonad. Moreover, these effects were suppressed by antioxidant treatment and were more severe in oxidative stress-sensitive *mev-1* mutants, confirming that gliadin induces oxidative stress responses in *C. elegans*, resulting in intestinal barrier disruption and increases in germ cell apoptosis. 

We observed that intestinal barrier disruption after gliadin intake increased in severity with age (Figure 5) and was suppressed by treatment with the antioxidant NAC (Figure 6). This finding supports an idea that intestinal ROS production triggered by gliadin intake causes the disruption of the intestinal barrier and suggests that the combination of oxidative stress induced by gliadin intake and aging accelerates gliadin-triggered intestinal disruption. To confirm that the intestinal disruption is specifically due to gliadin intake in a *C. elegans* model, we also treated worms with wheat gluten hydrolysate (WGH) and found that WGH does not cause intestinal disruption. WGH has been reported to have beneficial antioxidant effects on *C. elegans* [22]. In addition, similar effects to those of gliadin intake were observed when worms were fed with the synthetic gliadin peptides GP111–130, and GP151–170, suggesting that specific domains of α-gliadin are involved in gliadin-triggered effects (Figure 2 and Figure 6). Such domain-specific responses have been reported for celiac disease patients [7]. The effects of gliadin on the intestine observed in this study are supported by several previous studies reporting that the expression of apical junctional proteins is altered by gliadin intake [54,55]. Oxidative stresses caused by an increase in ROS production can disturb the balance of the physiological antioxidant systems [56]. It is possible that the oxidative stress induced by gliadin is responsible for free radical damage of important cellular structures, adversely affecting their functions [57,58,59]. It has been reported that gluten consumption by celiac disease patients induces the overproduction of ROS, triggering a cascade of reactions causing oxidative stress throughout the body [60]. It has also been suggested that gliadin disturbs the balance between cellular oxidants and antioxidants through the overproduction of ROS in the small intestinal mucosa of celiac patients [61]. Therefore, there seems to be a strong correlation between intestinal disruption caused by ROS production.

We also examined the relationship between gliadin intake and germ cell apoptosis in this study. Germ cell apoptosis can be induced in response to various stresses, including oxidative and osmotic stress, heat shock, and starvation [62]. We found that gliadin intake significantly increases the levels of germ cell apoptosis in *C. elegans*. Moreover, we found that GIGA depends on CEP-1 activity and MAPK signaling and is independent of DNA damage responses, as indicated by inactivation of HUS-1 after gliadin treatment since HUS-1 is activated in response to DNA damages [44]. We also examined tissue-specific requirements of *cep-1* and *mpk-1* through tissue-biased RNAi and found that *cep-1* is required in the germ line for GIGA, whereas *mpk-1* is required for GIGA in both the soma and the germ line. Similar results were obtained by treating *C. elegans* with the synthetic peptides GP111–130 and GP151–170. All these observed effects were suppressed upon co-treatment with the antioxidant NAC, indicating that ROS production by gliadin intake induces high levels of germ cell apoptosis (Figure 8 and Figure 10). Interestingly, we also observed that the oxidative-sensitive *mev-1* mutant is hypersensitive to gliadin, based on its level of GIGA, which was also suppressed by co-treatment with the antioxidant NAC. These observations indicate that the ROS generated in the intestine by gliadin intake induces increased germ cell apoptosis in the gonad and that this process requires CEP-1 activity in the germ line and MAPK signaling in the soma and germ line. Additionally, the specific somatic tissues in which MAPK signaling occurs for GIGA need to be determined. Previous studies have demonstrated that ROS can mediate the activation of the MAPK pathway [63,64]. We showed that gliadin treatment neither increases GIGA nor induces ROS production in *mpk-1* mutants (Figure 8A,E). It is thus possible that ROS-mediated signaling through the MAPK pathway is essential to trigger the effects of gliadin intake. Whether there are any connections between CEP-1 activity and MAPK signaling in germ cells remains to be investigated.

In summary, our results suggest that oxidative stress is strongly associated with gluten-related disorders. We demonstrated that ROS production in the intestine disrupts the intestinal barrier and triggers the germline responses. This inter-organ communication via disrupted intestinal integrity could be one reason why non-celiac gluten sensitivity (NCGS) displays pleiotropic symptoms in both the intestine and other organs, as showcased by NCGS-associated neuronal disorders. *C. elegans* is an excellent model for studying the involvement of neuronal signals in gliadin effects because of its well-understood neuronal network. Out of 959 somatic cells in *C. elegans*, 302 are neuronal cells whose connections are well defined [65]. Investigations to see if neuronal networks are involved in the response of *C. elegans* to gliadin intake in addition to the responses in the gonad would be an interesting future extension of this work.
